# Sann-Joong-Kuey-Jian-Tang induces autophagy in HepG2 cells via regulation of the phosphoinositide-3 kinase/Akt/mammalian target of rapamycin and p38 mitogen-activated protein kinase pathways

**DOI:** 10.3892/mmr.2015.3573

**Published:** 2015-03-31

**Authors:** WAN-LING CHUANG, CHIN-CHENG SU, PING-YI LIN, CHI-CHEN LIN, YAO-LI CHEN

**Affiliations:** 1 Transplant Medicine and Surgery Research Centre, Changhua Christian Hospital, Changhua 50006, Taiwan, R.O.C.; 2 Department of Surgery, Changhua Christian Hospital, Changhua 50006, Taiwan, R.O.C.; 3 Tumor Research Center of Integrative Medicine, Changhua Christian Hospital, Changhua 50006, Taiwan, R.O.C.; 4 Comprehensive Breast Cancer Center, Changhua Christian Hospital, Changhua 50006, Taiwan, R.O.C.; 5 College of Chinese Medicine, China Medical University, Taichung 40402, Taiwan, R.O.C.; 6 Institute of Biomedical Science, National Chung-Hsing University, Taichung 40227, Taiwan, R.O.C.; 7 Department of Medical Research and Education, Taichung Veterans General Hospital, Taichung 40705, Taiwan, R.O.C.; 8 Rong Hsing Research Center for Translational Medicine, National Chung Hsing University, Taichung 40227, Taiwan, R.O.C.; 9 School of Medicine, Kaohsiung Medical University, Kaohsiung 80708, Taiwan, R.O.C.

**Keywords:** autophagy, HepG-2 cells, Sann-Joong-Kuey-Jian-Tang, phosphoinositide 3-kinase, mammalian target of rapamycin, Akt, p38 mitogen-activated protein kinase

## Abstract

Sann-Joong-Kuey-Jian-Tang (SJKJT), a traditional Chinese medicine, was previously reported to induce autophagy and inhibit the proliferation of the human HepG2 hepatocellular carcinoma cell line via an extrinsic pathway. In the present study, the effects of SJKJT-induced autophagy and the cytotoxic mechanisms mediating these effects were investigated in HepG2 cells. The cytotoxicity of SJKJT in the HepG2 cells was evaluated using a 3-(4,5-dimethylthiazol-2-yl)-2,5-diphenyltetrazolium bromide assay. The results demonstrated that the half-maximal inhibitory concentration of SJKJT was 2.91 mg/ml at 24 h, 1.64 mg/ml at 48 h and 1.26 mg/ml at 72 h. The results of confocal fluorescence microscopy indicated that SJKJT resulted in the accumulation of green fluorescent protein-LC3 and vacuolation of the cytoplasm. Flow cytometric analysis revealed the accumulation of acidic vesicular organelles. Furthermore, western blot analysis, used to determine the expression levels of autophagy-associated proteins, demonstrated that the HepG2 cells treated with SJKJT exhibited LC3B-I/LC3B-II conversion, increased expression levels of Beclin, Atg-3 and Atg-5 and reduced expression levels of p62 and decreased signaling of the phosphoinositide-3 kinase/Akt/mammalian target of rapamycin and the p38 mitogen-activated protein kinase pathways. Taken together, these findings may assist in the development of novel chemotherapeutic agents for the treatment of malignant types of liver cancer.

## Introduction

Autophagy is a key intracellular pathway, which involves the degradation of damaged organelles and misfolded proteins through the actions of lysosomes (1). Autophagy is involved in cell survival and cell death, depending on the stimuli (2,3). Previous studies have demonstrated that certain plant- and animal-derived compounds are able to induce autophagy, indicating that they may possess potential anticancer properties (4,5). For example, resveratrol, a naturally occurring polyphenol in a number of plants, has been observed to induce autophagy in ovarian cancer cells and in human U373 glioma cells (6); curcumin induces autophagy by activating the Akt/mammalian target of rapamycin (mTOR)/p70S6 kinase and extracellular signal-regulated kinase (ERK)1/2 signaling pathways (7); and arenobufagin has been reported to induce apoptosis and autophagy in human hepatocellular carcinoma cells by inhibiting the phosphoinositide-3 kinase (PI3K)/Akt/mTOR pathway (8).

PI3K and Akt have been implicated in the activation of mTOR protein kinase. The PI3K/Akt/mTOR signaling pathway is a key regulator of a wide range of physiological cell functions, including proliferation, motility, differentiation, growth, survival, metabolism, autophagy and apoptosis (9).

The mitogen-activated protein kinase (MAPK) pathways have been observed to serve key functions in the development and progression of cancer (10). The three major MAPK pathways include the p38 MAPK pathway, the ERK1/2 (p44/p42) pathway and the c-Jun N-terminal kinase (JNK) pathway (11). Activation of the ERK1/2 pathway has been associated with cell survival, proliferation and differentiation, and the JNK pathway has been observed to regulate diverse biological functions, including cytoprotection, apoptosis and metabolism (10). Previous studies have indicated that JNK and p38 are activated by chemotherapeutic drugs, inflammatory cytokines and reactive oxygen species (ROS) (12,13).

Sann-Joong-Kuey-Jian-Tang (SJKJT), a traditional Chinese medicine, has been observed to inhibit the proliferation of MCF-7 and MDA-MB-231 human breast cancer cells by inhibiting the progression of the cell cycle and inducing apoptosis (14). SJKJT has also been found to induce apoptosis via upregulating the protein expression of microtubule-associated protein light chain 3 (15), Fas and tumor necrosis factor-α (TNF-α) (16) and upregulating the antitumor activity of 5-fluorouracil in colo 205 cells (17). It also reduces the protein expression levels of myeloid cell leukemia 1 and translationally controlled tumor protein, and upregulates the protein expression levels of TNF-α and B-cell-associated X protein (Bax) in pancreatic carcinoma cells (18). SJKJT contains several active ingredients, including baicalin, berberine, gentiopicroside, glycyrrhizin, palmatine, mangiferin and wogonin (19). Our previous study reported that SJKJT induces apoptosis in HepG2 cells by increasing the expression levels of TNF-α, caspase-8, caspase-3 and Bax (20). Although SJKJT has been demonstrated to induce autophagy in HepG2 cells, the underlying mechanism of action remains to be elucidated. In the present study, the molecular pathways through which SJKJT induces autophagy in the human HepG2 hepatocellular carcinoma cell line were investigated.

## Materials and methods

### Chemical reagents

The MTT [3-(4,5-dimethylthiazol-2-yl)-2,5-diphenyltetrazolium bromide], dimethyl sulfoxide (DMSO) and acridine orange were obtained from Merck Millipore (Darmstadt, Germany). Paraformaldehyde, Triton X-100 and propidium iodide (PI) were obtained from Sigma-Aldrich (St. Louis, MO, USA). Minimum essential medium (MEM-α), fetal bovine serum (FBS), 10X phosphate-buffered saline (PBS) and penicillin-streptomycin were obtained from Gibco Life Technologies (Grand Island, NY, USA). Lipofectamine 2000 transfection reagent and 4′6-diamidino-2-phenylindole (DAPI) were obtained from Invitrogen Life Technologies (Carlsbad, CA, USA). The 10X radioimmunoprecipitation (RIPA) lysis buffer was obtained from EMD Millipore (Billerica, MA, USA). Tween 20 was obtained from AMRESCO, Inc. (Solon, OH, USA). WesternBright Quantum enhanced chemiluminescence (ECL) horseradish peroxidase (HRP) was obtained from Advansta (Menlo Park, CA, USA).

### Cell culture

The human HepG2 liver carcinoma cell line was obtained from the Bioresource Collection and Research Center (Hsinchu, Taiwan). The cells were maintained in MEM-α medium with 10% FBS, 100 U/ml penicillin and 0.1 mg/ml streptomycin at 37°C in a humidified atmosphere of 95% air and 5% CO_2_.

### Preparation of SJKJT

SJKJT consists of 17 species of medicinal herbs, including *Glycyrrhiza uralensis* Fisch, *Coptis chinensis* Franch, *Cimicifuga heracleifolia* Komar, *Phellodendron amurense* Rupr, *Anemarrhena* asphodeloides Bunge, *Scutellaria baicalensis* Georgi, *Gentiana scabra* Bunge, *Trichosanthes cucumer oides* Maxim, *Platycodon grandiflour*, *Laminaria japonica* Aresch, *Bupleurum chinese* DC, *Curcuma aeruginosa* Roxb, *Sparganium stoloniferum* Bucch, *Forsythia suspense* Vahl, *Pueraria lobata* Ohwi, *Paeonia lactiflora* Pall and *Angelica sinensis* Diels (12). The crude extract of SJKJT used in the present study was obtained from Chuang Song Zong Pharmaceutical Co., Ltd. (Ligang Plant, Taiwan). The SJKJT was diluted in distilled sterilized PBS to create a stock solution (100 mg/ml), which was then stored at −20°C, according to the manufacture’s instructions. The final concentrations of SJKJT were 0.8, 1.6 and 2 mg/ml.

### Measurement of cell viability in the HepG2 cells

The cell viability was assessed using an MTT assay. The HepG2 cells were plated in a 96-well plate at a density of 2×10^4^ cells/well and were incubated overnight at 37°C. Subsequent to the removal of the MEM-α medium, the cells were treated with various concentrations (0.5, 1, 1.5, 2, 2.5 or 5 mg/ml) of SJKJT for 24, 48 or 72 h. Following treatment with SJKJT, the cells were treated with MTT (1 mg/ml) and were incubated for 2 h at 37°C. The medium was removed and the purple-blue MTT formazan precipitate was dissolved in 100 *µ*l DMSO. The absorbance was measured at a wavelength of 590 nm, with the results expressed as a percentage of the untreated controls. The percentage of proliferation was calculated using the following formula: Proliferation (%) = (ODtest − ODblank × 100, where ODtest and ODblank represent the optical density of the test substances and the blank controls, respectively.

### Acridine orange staining for the analysis of autophagy

Autophagy is characterized by the formation of acidic vesicular organelles (AVOs). To detect AVOs, cells can be stained with acridine orange, a nucleic acid-specific fluorescent cationic dye (21). The cells were seeded at a density of 2×10^5^ cells in six-well plates and allowed to attach. Subsequent to treatment with 0.8 mg/ml SJKJT for 6 h at 37°C, the cells were stained with 1 *µ*g/ml acridine orange for 10 min at 37°C, collected by trypsinization (Gibco Life Technologies) and resuspended in PBS. The green (510–530 nm) and red (650 nm) fluorescence, which was emitted from 1×10^4^ cells illuminated with blue (488 nm) excitation light, were measured using a BD accuri C5 flow cytometer and BD Accuri™ C6 version 1.0.264.21 software (BD Biosciences, Franklin Lakes, NJ, USA).

### Green fluorescent protein (GFP-LC3) plasmid transfection

HepG2 cells (1×10^5^) were seeded onto six-well plates and transfected with a GFP-LC3 expression plasmid (kind gift from Dr Lin, Institute of Biomedical Science, National Chung-Hsing University, Taichung, Taiwan) using Lipofectamine 2000 transfection reagent. Following transfection for 24 h at 37°C, the cells were treated with 0.8 mg/ml SJKJT or 2 *µ*g/ml rapamycin (EMD Millipore) for 12 h at 37°C. The cells were then fixed with 4% paraformaldehyde for 30 min at 37°C and washed twice in PBS. The cell nuclei were then counterstained with 1 mg/ml DAPI and images of the cells were captured from four non-overlapping fields using a Leica SP5 confocal laser-scanning microscope (Leica Microsystems GmbH, Wetzlar, Germany).

### Nuclei PI staining analysis

The HepG2 Cells (1×10^5^) were plated onto 12-well plates and treated with SJKJT (0.8 mg/ml) for four 0, 3, 6 or 12 h. The cells were then fixed with 4% formaldehyde for 30 min at room temperature, and were washed twice with PBS. The cells were then permeabilized in 0.25% Triton-X 100 for 10 min at room temperature and then washed three times in PBS. The nuclei were stained using PI (5 *µ*g/ml) for 10 min and were then examined under an Olympus IX81 microscope (Olympus, Tokyo, Japan).

### Cell lysis and western blot analysis

Following SJKJT treatment, the HepG2 cells were washed with ice-cold PBS. The cells were lysed in 1X RIPA lysis buffer, containing protease inhibitors. The cells were then removed and collected into eppendorf tubes (Quality Scientific Plastics, Inc., San Diego, CA, USA), which were agitated for 30 min at 4°C, followed by centrifugation at 13,000 × g for 10 min at 4°C (5415D; Eppendorf, Hamburg, Germany). The protein concentrations were determined using a Bicinchoninic Acid Protein Assay kit (Thermo Fisher Scientific, Waltham, MA, USA). Equal quantities of sample (10 *µ*g/lane) were loaded into wells containing 6–10% SDS-polyacrylamide gel (Bio-Rad Laboratories, Inc., Hercules, CA, USA), and were separated by SDS-PAGE. The separated proteins were then electrophoretically transferred onto polyvinylidene difluoride membranes (EMD Millipore) at 400 mA for 2 h. The membranes were then incubated in blocking buffer (PBS with 0.05% Tween 20 and 5% non fat dry-milk) for 1 h at room temperature, followed by incubation with the following primary antibodies overnight at 4°C: Rabbit polyclonal Beclin-1 (cat. no. 3738); rabbit polyclonal LC3B (cat. no. 2775); rabbit monoclonal p62 (cat. no. 8025); rabbit polyclonal phosphorylated (p)-PI3K (cat. no. 4228); rabbit polyclonal PI3K (cat. no. 4292); rabbit polyclonal p-mTOR (cat. no. 2971); rabbit monoclonal mTOR (cat. no. 2983); rabbit polyclonal p-Akt (cat. no. 9275); rabbit polyclonal Akt (cay. no. 9272); rabbit monoclonal p-ERK1/2 (cat. no. 4370); rabbit monclonal ERK1/2 (cat. no. 4695); rabbit monoclonal p-SAPK/JNK (cat. no.4668); rabbit polyclonal SAPK/JNK (cat. no. 9252); rabbit monoclonal p-p38 (cat. no. 4511); rabbit polyclonal p38 (cat. no. 9212) (all Cell Signaling Technology, Inc., Danvers, MA, USA); rabbit monoclonal Atg-3 (cat. no. GTX63041); and rabbit monoclonal Atg-5 (cat. no. GTX62601; both GeneTex, Inc., Irvine, CA, USA) and mouse monoclonal β-actin (cat. no. A5441; Sigma-Aldrich. All primary antibodies were used at 1:1,000 dilutions. The membranes were then incubated with HRP-conjugated goat anti-rabbit (1:10,000; cat. no. AP132P) and goat anti-mouse (1:10,000; cat. no. AP124P) secondary antibodies (Merck Millipore) for 1 h at room temperature. The blots were washed three times in 1X PBS-Tween 20 solution and incubated for 1 min with WesternBright Quantum enhanced chemiluminescence reagents. The results were visualized by exposing the blots to Super RX-N film (Fujifilm Corporation, Tokyo, Japan). The protein expression levels were quantified using Image J software (1.42q; National Institutes of Health, Bethesda, MD, USA, 2009).

### Statistical analyses

The data are expressed as the mean ± standard deviation, and were compared using Student’s t-test. P<0.05 was considered to indicate a statistically significant difference. All statistical analyses were performed using GraphPad Prism software, version 4.0 (GraphPad Software, Inc., La Jolla, CA, USA).

## Results

### Treatment with SJKJT inhibits the proliferation of HepG2 cells

**Figure 1:**
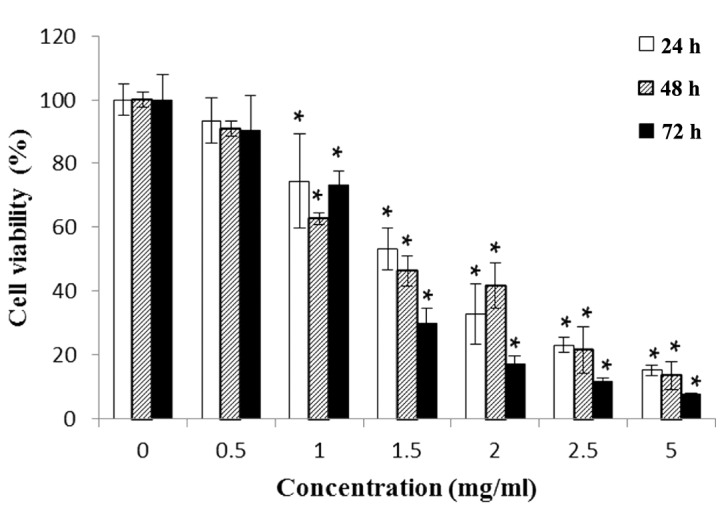
HepG2 cells (2×10^4^ cells/well) treated with various concentrations of SJKJT (0, 0.5, 1, 1.5, 2, 2.5 or 5 mg/ml) for 24, 48 or 72 h. Cell viability was measured using a 3-(4,5-dimethylthiazol-2-yl)-2,5-diphenyltetrazolium bromide assay. The cytotoxicity of SJKJT in the HepG2 cells was dose-dependent. The data are expressed as the mean ± standard deviation of three experiments. ^*^P<0.001, vs. control (0 mg/ml). SJKJT, Sann-Joong-Kuey-Jian-Tang.

The HepG2 cells were treated with various concentrations of SJKJT (0, 0.5, 1, 1.5, 2, 2.5 or 5 mg/ml) for 24, 48 and 72 h and cell viability was measured using an MTT assay. The half-maximal inhibitory concentration (IC_50_) was 2.91 mg/ml at 24 h, 1.64 mg/ml at 48 h and 1.26 mg/ml at 72 h, thus a dose-dependent reduction in proliferation was observed with the administration of SJKJT (Fig. 1).

### SJKJT induces autophagy in HepG2 cells

**Figure 2:**
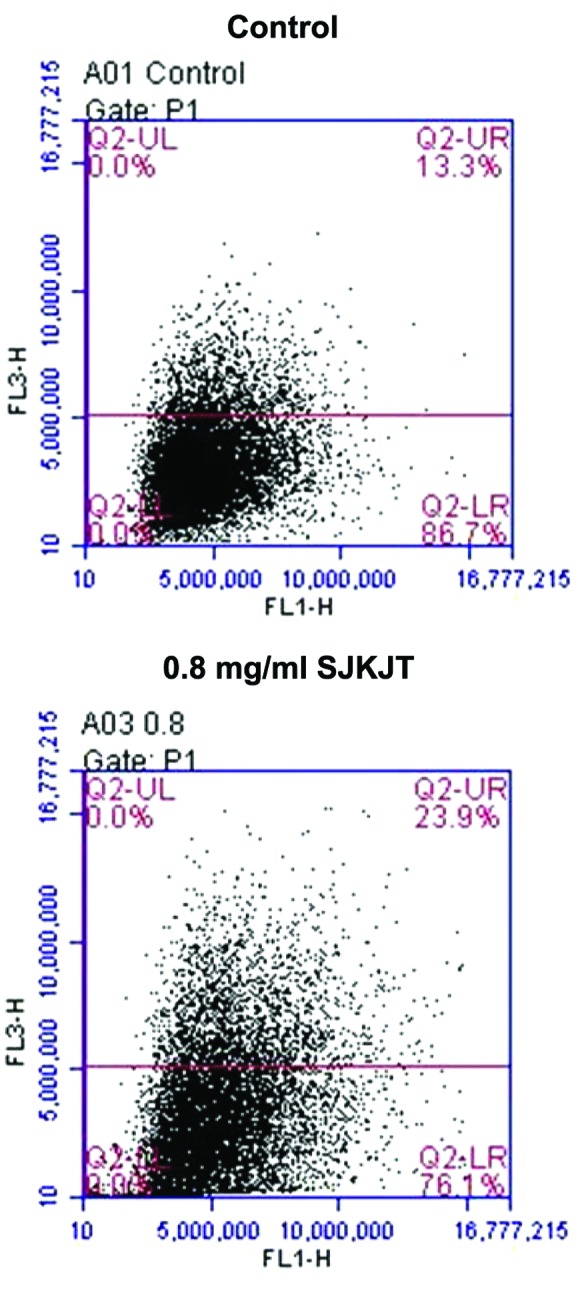
SJKJT induces autophagy in HepG2 cells. The cells were treated with 0.8 mg/ml SJKJT for 6 h and analyzed by flow cytometry to measure the production of acidic vesicular organelles. SJKJT, Sann-Joong-Kuey-Jian-Tang.

**Figure 3:**
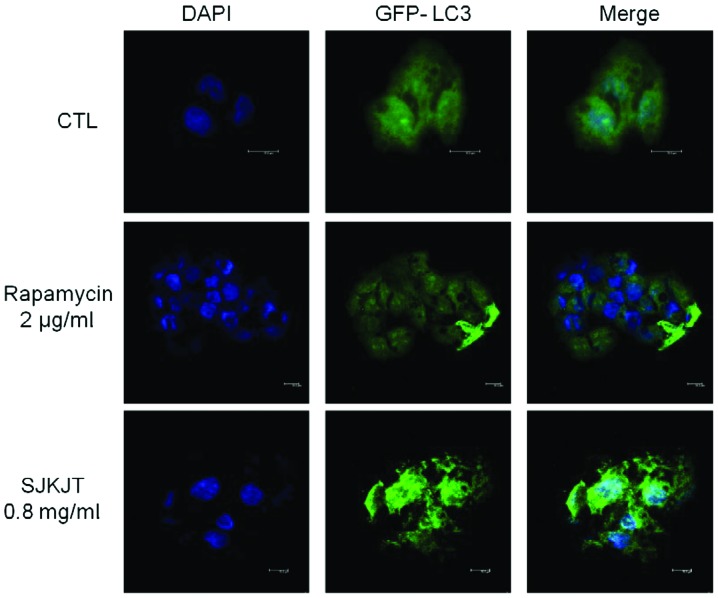
Confocal fluorescence microscopy used to detect the levels of GFP-LC3 in SJKJT-treated HepG2 cells. The cells were transfected with GFP-LC3, followed by treatment with 0.8 mg/ml SJKJT or 2 *µ*g/ml rapamycin for 12 h, and compared with the untreated control. Nuclei were stained with DAPI, as shown in blue (magnification, ×63). GFP, green fluorescent protein; DAPI, 4′6-diamidino-2-phenylindole; SJKJT, Sann-Joong-Kuey-Jian-Tang; CTL, untreated control.

The HepG2 cells were treated with 0.8 mg/ml SJKJT for 6 h, stained with 1 *µ*g/ml acridine orange, and examined by flow cytometry. The results demonstrated that exposure to 0.8 mg/ml SJKJT for 6 h was effective at inducing autophagy in the HepG2 cells (Fig. 2). Subsequently, GFP-LC3 plasmids and DAPI staining were used to observe the efficiency of autophagosome/lysosome fusion in cells treated with or without 0.8 mg/ml SJKJT for 12 h. The numbers of GFP-LC3B-labled puncta in the cytosol were markedly higher in the SJKJT group compared with the control group (Fig. 3).

### PI nuclear staining detects morphological alterations in HepG2 cells

**Figure 4:**
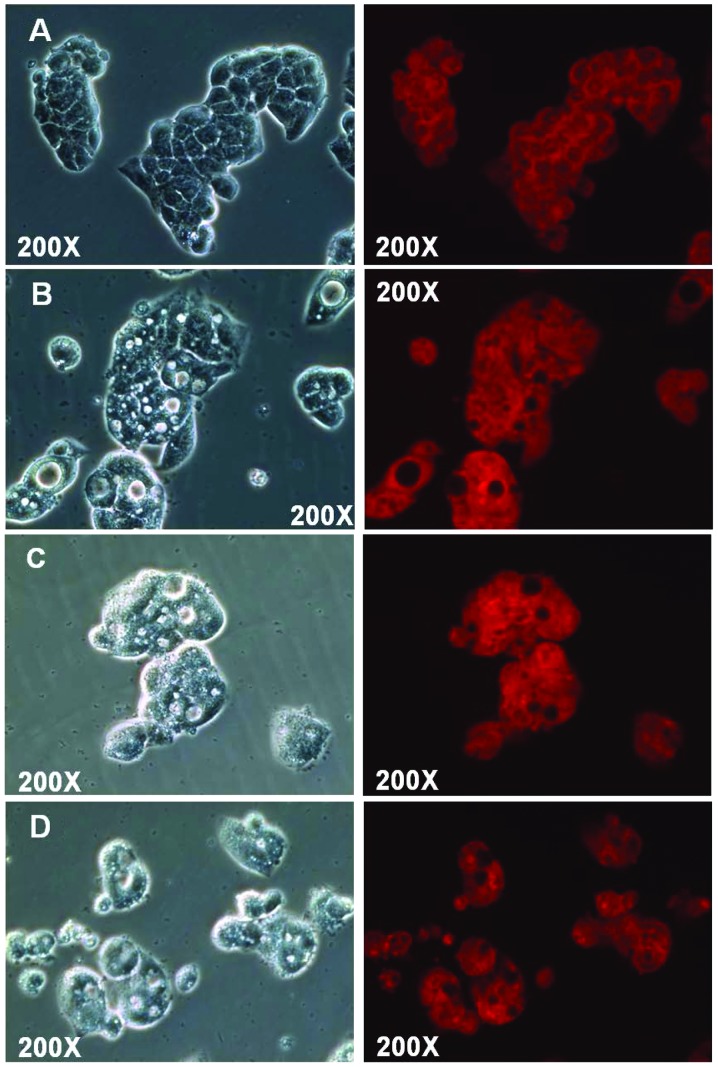
Induction of autophagy by SJKJT on vacuolation of the cytoplasm in HepG2 cells. Morphological changes in the HepG2 cells, following treatment with 0.8 mg/ml SJKJT for (A) 0, (B) 3, (C) 6 or (D) 12 h, were observed under a phase-contrast microscope (magnification, ×200). SJKJT, Sann-Joong-Kuey-Jian-Tang.

The HepG2 cells were treated with 0.8 mg/ml SJKJT for different durations (0, 3, 6 and 12 h) and were then fixed with 4% paraformaldehyde. Following permeabilization of the cell membranes, the nuclei were stained with PI (5 *µ*g/ml) in order to detect morphological alterations in the HepG2 cells. The results indicated that the number of cells with a vacuolated cytoplasm was markedly higher in the SJKJT-treated group compared with the control group (Fig. 4).

### SJKJT alters the levels of autophagy-associated proteins in HepG2 cells

**Figure 5:**
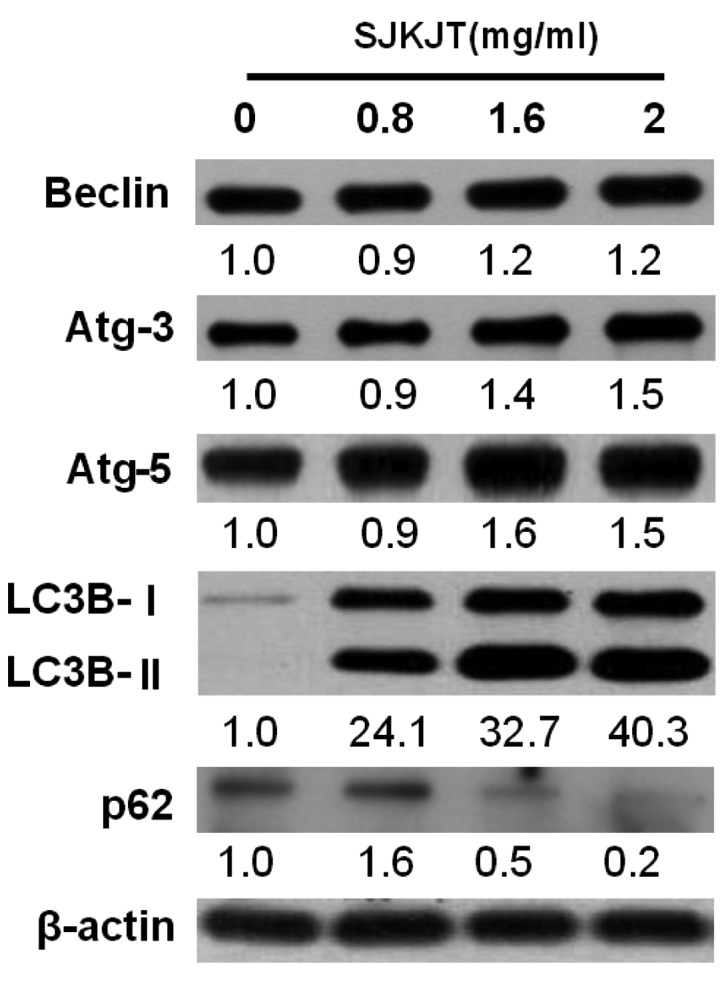
Effects of SJKJT on the expression levels of the Beclin, Atg3, Atg5, LC3B-II and p62 autophagic indicators in HepG2 cells by western blot analysis. The cells were treated with various concentrations of SJKJT (0, 0.8, 1.6 or 2 mg/ml) for 24 h. β-actin served as a loading control. SJKJT, Sann-Joong-Kuey-Jian-Tang.

**Figure 6:**
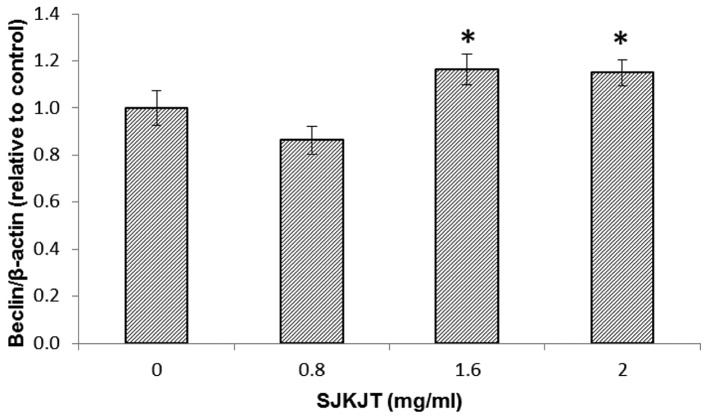
Protein expression of Beclin in the HepG2 cells. Data are expressed as the mean ± standard deviation of three experiments. ^*^P<0.001, vs. control (0 mg/ml). SJKJT, Sann-Joong-Kuey-Jian-Tang.

**Figure 7:**
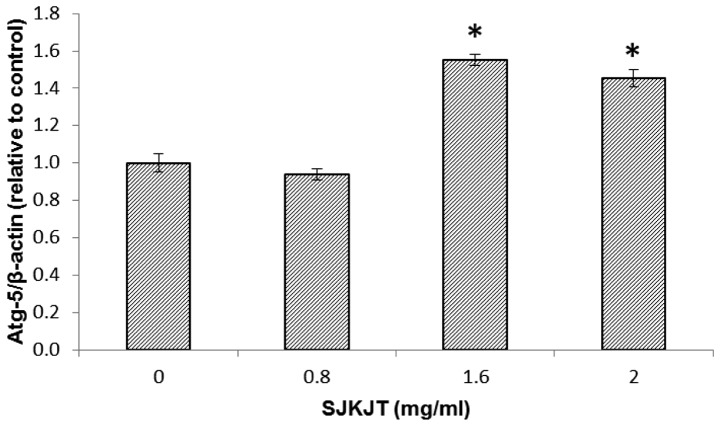
Protein expression of Atg5 in the HepG2 cells. Data are expressed as the mean ± standard deviation of three experiments. ^*^P<0.001, vs. control (0 mg/ml). SJKJT, Sann-Joong-Kuey-Jian-Tang.

Western blot analysis was performed to detect changes in the expression levels of the Beclin, Atg-3, Atg-5, LC3B-II and p62 autophagy-associated proteins in the HepG2 cells following exposure to various concentrations of SJKJT (0, 0.8, 1.6 and 2 mg/ml) for 24 h. The results revealed that the expression levels of Beclin, Atg-3, Atg-5 and LC3B-II were significantly increased (P<0.001) and expression levels of p62 were significantly reduced (P<0.001) following treatment with SJKJT at 0.8 mg/ml (Fig. 5). Figs. 6 and 7 indicate the protein expression of Beclin and Atg-5 in the HepG2 cells, respectively following treatment with SJKJT (0, 0.8, 1.6 or 2 mg/ml). The results revealed that the expression levels of Beclin and Atg-5 were significantly increased (P<0.001) following treatment with SJKJT at 1.6 and 2 mg/ml.

### SJKJT induces autophagy in HepG2 cells by upregulating the PI3K/Akt/mTOR pathway

**Figure 8:**
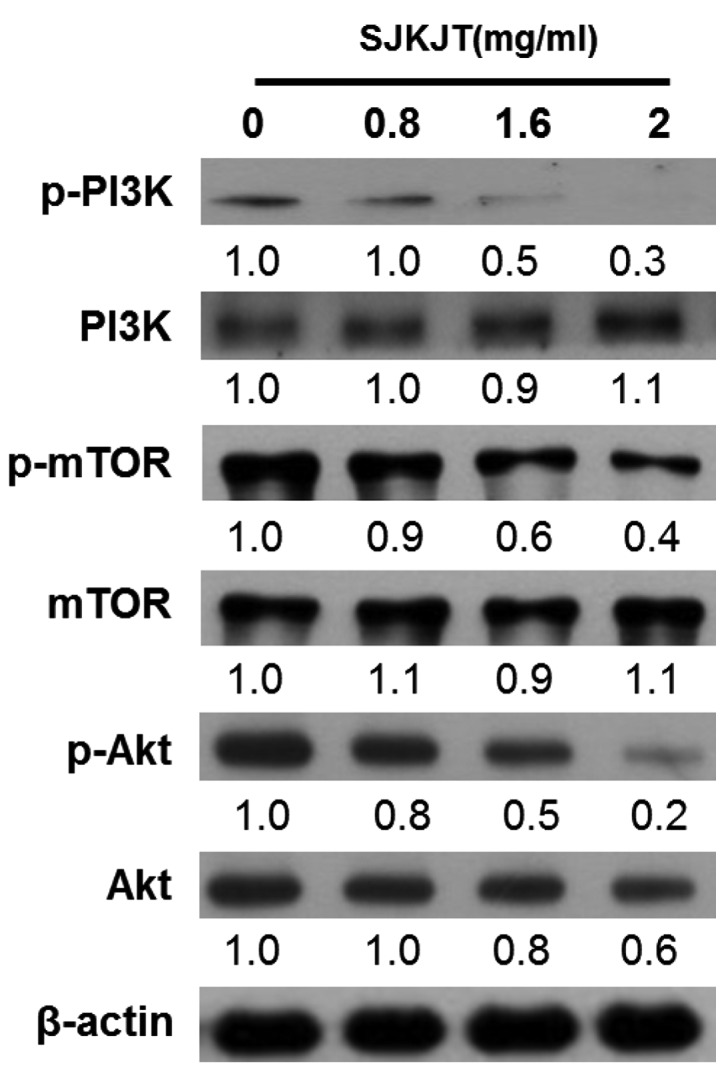
Effects on SJKJT on the PI3K/Akt/mTOR pathway. The cells were treated with various concentrations of SJKJT (0, 0.8, 1.6 or 2 mg/ml) for 24 h and the expressions of p-PI3K, PI3K, mTOR, p-mTOR, p-Akt and Akt in HepG2 cells were analyzed by western blot analysis. β-actin served as a loading control. SJKJT, Sann-Joong-Kuey-Jian-Tang; PI3K, phosphoinositide-3 kinase; mTOR, mammalian target of rapamysin; p-, phosphorylated.

The PI3K/Akt/mTOR signaling pathway is a well-known survival pathway, involved in the regulation of cell growth, tumorigenesis and the cell cycle (22). Western blot analysis was performed to measure changes in protein expression levels of p-PI3K, PI3k, p-mTOR, mTOR, p-Akt and Akt in the HepG2 cells following treatment with various concentrations of SJKJT (0, 0.8, 1.6 and 2 mg/ml) and in vehicle-treated control cells after 24 h. The results demonstrated that the expression levels of p-PI3K, p-mTOR and p-Akt were significantly lower in the HepG2 cells than in the untreated control cells (Fig. 8).

### SJKJT induces autophagy in HepG2 cells by upregulating the ERK1/2 and JNK1/2MAPK pathways

**Figure 9:**
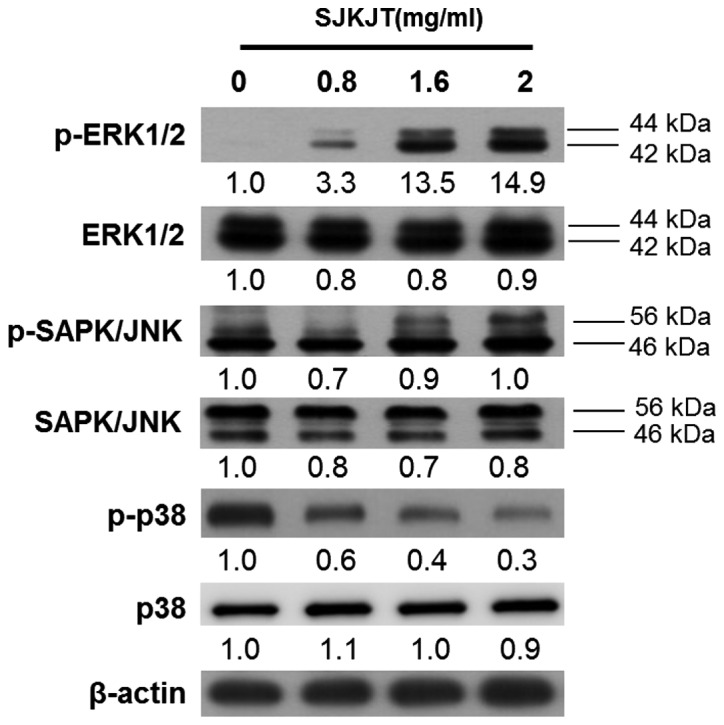
Identification of the association between the activation of MAPKs (ERK1/2 and JNK1/2) and the induction of autophagy following treatment with SJKJT in the HepG2 cells. The cells were treated with various concentrations of SJKJT (0, 0.8, 1.6 or 2 mg/ml) for 24 h. The activation of the MAPKs was determined through detection of the levels of phosphorylated protein by western blot analysis. β-actin served as a loading control. MAPK, mitogen-activated protein kinase; ERK, extracellular signal-regulated kinase; JNK, c-Jun N-terminal kinase; SJKJT, Sann-Joong-Kuey-Jian-Tang; p-, phosphorylated.

MAPK signaling is important in the outcome of, and sensitivity to, anticancer therapies (23). These activated kinases transmit extracellular signals, which regulate cell proliferation, growth, differentiation, migration and apoptosis (24). To examine whether SJKJT activates the ERK1/2 and JNK1/2 MAPK pathways in HepG2 cells, western blot analysis was performed to detect the expression levels of p-ERK1/2, ERK1/2, p-JNK, JNK, p-p38 and p38. It was observed that, following treatment of the HepG2 cells with various concentrations of SJKJT (0, 0.8, 1.6 and 2 mg/ml) for 24 h, the expression levels of p-ERK1/2 increased and that of p-p38 was reduced (Fig. 9).

## Discussion

SJKJT, a traditional Chinese medicine consisting of 17 species of medicinal herbs, has been demonstrated to exhibit antitumor and antiproliferative effects (15,17,25). In our previous study, SJKJT was observed to induce apoptosis in HepG2 cells by increasing the expression levels of TNF-α, caspase-8, caspase-3 and Bax (20). In the present study, SJKJT was observed to induce autophagy, via a mechanism involving the PI3K/Akt/mTOR and p38 MAPK pathways, and inhibit the proliferation of HepG2 cells in a time- and dose-dependent manner (Fig. 1).

Several chemotherapeutic agents have been demonstrated to induce autophagy in human hepatocellular carcinoma cells (26), including matrine and bufalin (27). Certain anticancer therapeutic agents have been identified to target pathways involved in autophagy, including dihydroartemisinin, which is reported to inhibit the nuclear translocation of nuclear factor-κB (28); thiazolidinedione, which induces autophagy in breast cancer cells by activating peroxisome proliferator-activated receptor-γ (29); curcumin, which suppresses the growth of malignant gliomas by inducing autophagy through a mechanism mediated by the Akt and ERK signaling pathways (30); and E platinum, which induces autophagy by inhibiting the phosphorylation of mTOR in BGC-823 gastric carcinoma cells (31). These results suggested that basal autophagy is crucial in the suppression of spontaneous tumorigenesis.

Autophagy has been observed to serve a key function in tumor suppression (32) and previous studies have indicated the inhibition of autophagy as a promising target for cancer therapy (33,34). A number of signaling pathways are involved in autophagy, including the class I PI3K/Akt/mTOR pathway (35). The results of the present study indicated that SJKJT inducedcell death by inhibiting the activation of PI3K in the HepG2 cells (Fig. 8). The inhibition of PI3K also resulted in the downregulation of p-mTOR, an essential protein for the induction of autophagy (Fig. 8). In addition, JNK activation has been found to be involved in the regulation of autophagy and apoptosis (36). The results of the present study demonstrated that SJKJT induced autophagy in the HepG2 cells via activation of the MAPK signaling pathways, including the ERK1/2 pathways (Fig. 9).

The association between autophagy and apoptosis has been widely investigated. Several pathways have been demonstrated to be involved in the regulation of autophagy and apoptosis, and the induction of autophagy-associated genes, including LC3-II, which is localized to preautophagosomes and autophagosomes (37), B-cell lymphoma (Bcl-2) and Bcl-extra large oncogenic proteins (38) and the induction of ROS (12). In the present study, treatment of HepG2 cells with SJKJT resulted in the formation of autophagosomes, accumulation of AVOs (Fig. 2), increase in cytoplasmic puncta (Fig. 3), increased protein expression of LC3-II and reduced expression of p62 (Fig. 5), indicating that SJKJT induced autophagy in the HepG2 cells.

In conclusion, the present study is the first, to the best of our knowledge, to demonstrate that SJKJT may induce autophagy and inhibit cell growth, by regulation of the PI3K/Akt/mTORand p38 MAPK pathways in HepG2 cells.
